# Genome Sequence of *Mannheimia haemolytica* Serotype 1 Strain 16041065 BH

**DOI:** 10.1128/genomeA.01721-16

**Published:** 2017-04-06

**Authors:** Sahlu Ayalew, Anthony W. Confer, Richard D. Hansen, Matthew Brian Couger

**Affiliations:** a Department of Veterinary Pathobiology, Center for Veterinary Health Sciences, Oklahoma State University, Stillwater, Oklahoma, USA; b SolidTech Animal Health Inc., Newcastle, Oklahoma, USA; c Oklahoma State University High Performance Computing Center, Stillwater, Oklahoma, USA

## Abstract

Here, we report the genome sequence of *Mannheimia haemolytica* serotype 1 strain 16041065 BH, which was recently isolated from a Midwestern calf that died due to *Mannheimia haemolytica*–induced pneumonia. This genome comprised a total of 2.7 Mb, with an *N*_50_ of 122 kb, and maintained hemolytic activity when grown on blood heart infusion agar supplemented with 5% sheep’s blood.

## GENOME ANNOUNCEMENT

Bovine respiratory disease complex (BRDC) costs the beef industry approximately one billion dollars a year in the United States due to mortality, morbidity, and the cost of antibiotics for treatment of animals that have been affected (1). BRDC is the result of the concerted action of numerous viruses and bacterial species, including bovine respiratory syncytial virus, parainfluenza 3, adenovirus, bovine viral diarrhea virus, and infectious bovine rhinotracheitis. Bacterial species include *Pasteurella multocida*, *Mannheimia haemolytica*, *Histophilus somni*, and *Mycoplasma bovis*. Studies suggest that it is the interaction of these pathogens and the failure of the animal’s immune system that produce full-blown disease (1–3). These same sources point to the possibility that bacterial pathogens cause the acute syndrome by invading the bovine respiratory tract that has been compromised by viral infections, environmental conditions, and/or other stress factors such as comingling, dehydration, and crowding during shipping (4, 5).

The bacterium that is most commonly associated with acute, severe respiratory disease is *Mannheimia haemolytica*. The disease usually strikes within the first 2 weeks after cattle have been shipped or stressed, and lesions are primarily a severe fibrinous pleuropneumonia (6). Understanding the nature of this bacterium, at both the cellular and molecular levels, is a prerequisite to developing efficacious treatment and prevention protocols. It is in light of this that we sequenced the genome of a recent isolate from a Midwestern calf that died due to *M. haemolytica*–induced pneumonia.

A well-isolated β-hemolytic colony was transferred into brain heart infusion broth, where it was grown to late log phase in a shaker incubator (37°C, 150 rpm). *M. haemolytica* cells were harvested by centrifugation (4°C, 12,000 × *g* for 20 min). A Wizard Genomic DNA purification kit (Promega, Madison, WI, USA) was used to extract genomic DNA. The purity and integrity of the genomic DNA data were determined by its 260/280 ratio on a nanodrop and gel electrophoresis on 1% agarose of both uncut and *EcoR*I-digested aliquots of the prep.

Reads were quality-filtered with standard Illumina filtering, and all quality-filtered reads were assembled using the short-read de Brujin graph assembly (6) program Velvet (7) with the settings of a *k*-mer value of 77 and a minimum contig coverage value of 7×. Gene models were created using the prokaryotic gene-calling software Prodigal (8). The Velvet assembly had a total size of 2,676,370 bp, 61 scaffolds, and a scaffold *N*_50_ of 122 kb and contained 2,583 predicted proteins. All gene models were annotated using a combination of homology comparison, domain prediction, and annotation in the public sequence databases. Programs used for functional annotation were an NCBI BLAST C++ homology search (9) and HMMER version 3.0 hmmscan (10) against the Pfam version 26.0 database (11). All genes were annotated for functional significance using the UniProt database (12).

### Accession number(s).

This whole-genome shotgun project has been deposited in GenBank under the accession number MLYL00000000. The version described in this paper is the first version, MLYL01000000.
